# Novel Trajectories Towards Possible Effects of Semaglutide for Amelioration of Reserpine-induced Fibromyalgia in Rats: Contribution of cAMP/PKA/p-CREB and M1/M2 Microglia Polarization

**DOI:** 10.1007/s11481-025-10196-4

**Published:** 2025-04-17

**Authors:** Mena Z. Shafiek, Hala F. Zaki, Ahmed F. Mohamed, Weam W. Ibrahim

**Affiliations:** 1 Department of Pharmacology and Toxicology, Faculty of Dentistry, Misr International University, Cairo, Egypt; 2 Department of Pharmacology and Toxicology, Faculty of Pharmacy, Cairo University, Cairo, 11562 Egypt; 3 Faculty of Pharmacy, King Salman International University (KSIU), South Sinai 46612, Sinai, Egypt

**Keywords:** Fibromyalgia, Semaglutide, Reserpine, CAMP/PKA/p-CREB and M1/M2 macrophage polarization

## Abstract

Fibromyalgia (FM) is a pain disorder characterized by pervasive musculoskeletal pain associated with exhaustion, depression, and irregular sleep patterns. Semaglutide, an innovative glucagon-like peptide-1 (GLP-1) agonist, has shown analgesic effects by modulating pain hypersensitivity in animal models of inflammatory pain. The objective of this study is to ascertain semaglutide's therapeutic potential against FM-like symptoms caused by reserpine. Reserpine (1 mg/kg/day; SC) was administered into rats for 3 consecutive days, then they were treated daily with semaglutide intraperitoneally in low (5 nmol/kg), intermediate (10 nmol/kg), or high doses (20 nmol/kg), respectively, for 14 consecutive days. Semaglutide alleviated reserpine induced histopathological and immunohistopathological changes in spinal cord of rats evidenced by a remarkable rise in immuno-expression of cluster of differentiation 163 (CD163) contrary to a significant diminution in CD86 level as compared with reserpine group. Semaglutide also had an analgesic effect and improved motor incoordination, and depression brought on by reserpine. Furthermore, it had an anti-inflammatory impact via stimulating cyclic adenosine monophosphate (cAMP)/ protein kinase A (PKA)/ cAMP response element (CRE)-binding protein (CREB) signaling pathway and shifting M1/M2 macrophage polarization towards the M2. Semaglutide's anti-inflammatory actions were manifested through inhibition of inducible nitric oxide synthase and reduction in dorsal root ganglia concentrations of tumor necrosis factor-α together with elevation in the levels of arginase-1 and interleukin-4.

**Graphical Abstract:**

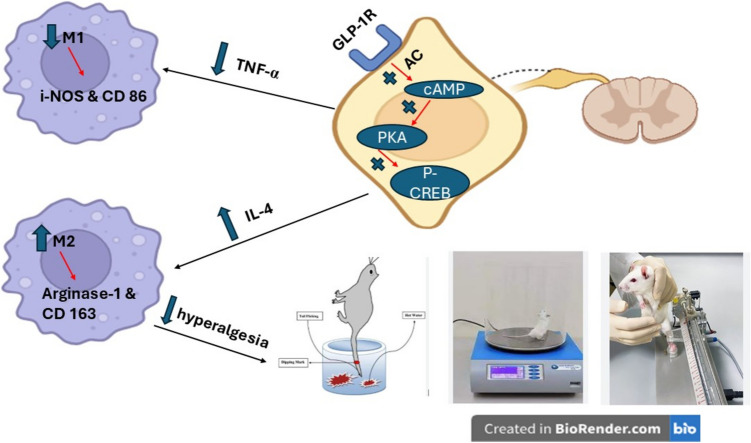

**Supplementary Information:**

The online version contains supplementary material available at 10.1007/s11481-025-10196-4.

## Introduction

Fibromyalgia (FM) is a medical syndrome typified by constant and pervasive musculoskeletal pain. Stiffness in the muscles and joints, irregular sleep patterns, fatigue, mood swings, cognitive impairment, anxiety, sadness, and the incapacity to operate everyday activities are the primary indications of this illness (Siracusa et al. 2021). Additionally, FM may be associated with certain diseases, including diabetes, rheumatic diseases, infections, and neurological or psychiatric conditions (Bellato et al. 2012). The primary alterations noted in FM include monoaminergic neurotransmission dysfunctions, which result in increases in excitatory neurotransmitters like substance P and glutamate and decreases in spinal cord serotonin and norepinephrine levels at the descending anti-nociceptive pathway level. Other abnormalities include altered endogenous brain opioid activity and dysregulation of dopamine. When combined, these findings appear to provide an explanation for the core pathophysiology of FM **(**Stahl 2009**)**.

A previous study discovered that because of the connection between insulin resistance (IR) and FM, there is a link between FM and diabetic mellitus (DM) (Lichtenstein et al. 2018). The undesirable lipid imbalance and the persistent increase in blood glucose levels caused by IR may damage the blood–brain barrier (BBB) and increase its permeability. In response to BBB damage, microglia are activated causing the release of cytokines and the subsequent neuroinflammatory cascades (Kopp et al. 2023). Neuroinflammation, which is mediated by microglia activation in the spinal cord, is crucial for developing the central sensitization in FM (Atta et al. 2023). In turn, neuroinflammation further exacerbates the BBB's destruction and increases its permeability leads to a vicious cycle of neuroinflammation (Shah and DeSilva 2012; Van Dyken and Lacoste 2018).

L cells in the small intestine produce the incretin hormone glucagon-like peptide-1 (GLP-1) in reaction to food consumption. It works by suppressing hunger, regulating blood sugar, and promoting good insulin signaling through its target receptor, the GLP-1 receptor (GLP-1R), which is present on β-cells in the pancreas. Consequently, many GLP-1R stimulating medications have been created and authorized for the management of type 2 DM via the US Food and Drug Administration and associated international regulatory organizations (Andreadis et al. 2018). Furthermore, expression of GLP-1Rs have been demonstrated in astrocytes, microglia, and neurons in several important brain areas such as arcuate nucleus, paraventricular nucleus and dorsal medial nucleus of the hypothalamus (Cui et al. 2022) and drugs that stimulate GLP-1R have demonstrated capability for being repurposed as a therapy for neurodegenerative conditions. since they have displayed anti-inflammatory, antiapoptotic, neurotrophic, as well as neuroprotective impacts in experimental models of neurodegenerative diseases (Li et al. 2010; Gilman et al. 2003; Li et al. 2021; Athauda & Foltynie 2018; Poupon-Bejuit et al. 2022; Kopp et al. 2023). Semaglutide, an innovative GLP-1 agonist, activates adenylate cyclase and subsequently increases cyclic adenosine monophosphate (cAMP), which stimulates exchange protein (Epac), producing antiapoptotic and anti-inflammatory actions (Tamayo-Trujillo et al. 2024). cAMP activates protein kinase A (PKA), that leads to stimulation and phosphorylation of the transcription factor cAMP response element (CRE)-binding protein (CREB) and also stimulates the mitogen activated protein kinase/extracellular signal-regulated kinase (MAPK/ERK) anti-inflammatory cascade (Jolivalt et al. 2011; Tamayo-Trujillo et al. 2024). As compared to liraglutide and exenatide, semaglutide has longer half lifetime owing to its higher albumin binding and longer fatty acids in its structure, in addition to possessing higher potency to GLP-1R (Knudsen and Lau 2019). Thus, the objective of this study is to ascertain the potential hypoalgesia and anti-inflammatory benefits of semaglutide, a GLP-1R agonist, in experimentally induced FM with an emphasis on amendment of cAMP/PKA/p-CREB pathway as contributing mechanisms to semaglutide’s effects.

## Materials and Methods

### Animals

Wistar male rats of approximately 3 months age, weighing 150 ± 20 g, were acquired from the house of animals at Faculty of Pharmacy, Cairo University, Cairo, Egypt. Throughout the experiment duration, animals were housed in controlled environments with temperatures of 22 ± 2 °C, humidity of 50–70%, as well as light/dark cycle of 12:12 h. One week preceding the trial techniques, they were given an accommodation period, and they also had full accessibility to water ad libitum and rat food.

Herein, male rats were employed to examine the influence of semaglutide without any interference by female rats’ estrous cycle and hormones, particularly that reserpine administration induces FM equally in both male and female rats (Nagakura et al. 2009). In addition, prior studies investigated several therapeutic interventions for FM in male rats (Kiso et al. 2018; Yao et al. 2019; Fusco et al. 2019).

### Compliance with Ethical Standards

The research project was reviewed and approved by Cairo University's Faculty of Pharmacy's Ethics Committee of Animal Care and Use under Permit number 3458. All investigative techniques were carried out according to the recommendations provided by the US National Institutes of Health's Guide for the Care and Use of Laboratory Animals (NIH publication No. 85–23, revised 2011) and the ARRIVE guidelines. All efforts were made to lessen animal pain.

### Drugs and Chemicals

Reserpine was purchased from Sigma-Aldrich Chemical Co. (St. Louis, MO, USA), while semaglutide was purchased from Novo nordisk company. In distilled water with 0.5% acetic acid, reserpine was dissolved, while semaglutide was dissolved in saline. The purest and highest analytical quality was used for all chemicals.

### Experimental Design

Forty male rats were haphazardly allocated into 5 groups (*n = *8/group). Group I (Control group): Rats were given subcutaneously injections of 0.5% acetic acid in distilled water for a period of 3 days, afterwards saline was intraperitoneally injected for 14 days. Group II: reserpine group (Res group): reserpine was given subcutaneously to rats at a dose of 1 mg/kg/day (Yao et al. 2020) for 3 days, then they were injected with saline intraperitoneally for 21 days. Group III: reserpine + low dose of semaglutide (Res + LD of Sema group), Group IV: reserpine + intermediate dose of semaglutide (Res + ID of Sema group), and Group V: reserpine + high dose of semaglutide (Res + HD of Sema): reserpine (1 mg/kg/day) was subcutaneously administered into rats for 3 successive days, then they were daily injected with intraperitoneal low (5 nmol/kg), intermediate (10 nmol/kg) (Yang et al. 2019), or high doses (20 nmol/kg) of semaglutide, respectively, for 14 consecutive days.

A 10 nmol/kg dose level of semaglutide was chosen depending on a previous study demonstrating its anti-inflammatory effects in a rat model of stroke (Yang et al. 2019). There are, however, no previous reports about the dose of amendment FM-like symptoms. So, LD of Sema and HD of Sema (5 and 20 nmol/kg) respectively were also chosen to make a dose response study to prove the most effective dose in mitigation of FM-like symptoms induced by reserpine.

Behavioral tests were carried out on 0, 4, 7, and 14 days, with a two-hour rest time in between each pair of behavioral assessments, which were made from least to most stressful (Yao et al. 2020). Once all behavioral tests have been completed on day 14, Rats were euthanized under phenobarbital anesthesia (40 mg/kg; intraperitoneally) (El-Sahar et al. 2020) by decapitation, and afterwards bilateral lumbar dorsal root ganglia (DRG) were isolated, saline-washed, dried and weighed (Cordaro et al. 2022; Ejiri et al. 2022). In every group, the samples of rats were assigned into two sets. The first set (*n = *3) involved handling lumbar DRG spinal cords for histological evaluation. The second subset's samples (*n = *5) were preserved at −80^◦^C for biochemical determinations after being frozen in liquid nitrogen. Depending on the results of behavioral tests and the histopathological examinations, high dose of semaglutide is chosen as the most effective dose to mitigate the FM-like changes induced reserpine. Thus, DRG samples of Res + HD of Sema group underwent biochemical assays to reveal the impact of semaglutide on the biochemical markers.

### Behavioral Tests

#### Tail Immersion Test

The purpose of the tail immersion test is to evaluate rodents' spinal heat sensitivity. The nociceptive reaction time, measured in seconds, was measured after sinking the end of the tail, 1 cm distal section, into a water bath that was kept at a steady 55 °C. The nociceptive endpoint was distinguished by either a swift flinch of the whole body of the rat or a severe shake of its tail. To prevent injury to the tissue, A cut-off period of 15 s was set (Deuis et al. 2017).

#### Rotarod Test

The test evaluates a rodent's motor coordination and balance. Prior to the experiments, rats were trained for two days in a row using a 5-lane rotarod device (Model 47750, UgoBasile, Italy) that is automated at an increasing velocity starting at 4 till 20 rpm and setting a cutoff time of 3 min for 3 runs daily. Rats chosen for this experiment were those that remained on the revolving rod for 3 min. Each rat was given three attempts of three minutes on the revolving rod on the day of the test. Every rat's average time to tumble from the revolving rod was observed (Deacon 2013).

#### Randall-Selitto Test

A widely used technique for evaluating mechanical hyperalgesia is the Randall-Selitto test. An analgesiometer “Ugo Basile, Italy, Model 7200” that produces a mechanical force gradually increasing on the mid-gastrocnemius muscle of that animal's hind limb was utilized to detect the threshold of hind limb withdrawal. To allow measuring the threshold, rats were rendered immobile by holding them using a soft cloth. To prevent tissue damage, the maximum cutoff load was 250 g (De la Luz-Cuellar et al. 2019).

#### Hot Plate Test

One of the most widely used methods for assessing rodent's supraspinal thermal nociception is the hot plate test, which is frequently utilized to evaluate thermal hyperalgesia. A hot plate “Ugo Basile, Italy, Model 7280” kept at 54–56 °C was used herein, on which the rats were placed. The latency taken to jump out of the enclosure or lick the hind paw was detected as the thermal pain behavior latency. A 12 s cutoff reaction time was selected to prevent physical harm (Deuis et al. 2017).

#### Forced Swimming Test

Depression is evaluated using the forced swimming test. Each rat was placed in a plastic cylinder with dimensions of 20 cm diameter and 50 cm heigh and filled with water (23–25 °C) at 30 cm deep so that they were unable to support themselves on the bottom of the cylinder. For every rat, a 5-min test was recorded on camera. The final four minutes of the test session were measured using a stopwatch to determine how long the subjects were immobile. When a rat ceased swimming and only made the slightest motions required to maintain its head over the water surface, it was deemed immobile (Yankelevitch-Yahav et al. 2015). Rats were trained for 10 min a day before performing the test.

### Histopathological Investigation

Lumber segments' DRG were preserved for 72 h in buffered formalin (10%). The samples were processed in a series of ethanol grades, then underwent xylene clearing, infiltration, and embedding in Paraplast tissue embedding media (Leica biosystems). Rotatory microtomes were used to cut 5 μm thick serial DRG tissue slices to show the DRG regions in various samples. These sections were then put on glass slides. Experienced histologists used Hematoxylin and Eosin, a common staining technique, to examine tissue sections under a blinded light microscope. All standard methodology and protocols for staining and fixing samples were carried out in accordance with (Culling 2013).

### Immunohistochemical Estimation of CD163 and CD86

Cluster of differentiation, CD86 and CD16, which are microglia surface markers of microphage 1 (M1, pro-inflammatory) and microphage 2 (M2, anti-inflammatory), consequently, were quantitively detected via immuno-histochemistry method observing the guidelines supplied by the manufacturer. Sections of deparaffinized recovered DRG tissue were blocked with 0.3% hydrogen peroxide for a period of 20 min and then incubated at 4 °C for the entire night with anti-CD163 primary antibody supplied by GeneTex, CA, USA (GTX35247, 1:100) as well as anti-CD86 primary antibody provided by Bioss, USA (bs-1035R, 1:150). Sections were cleaned using PBS and then incubated for 20 min with the HRP Envision kit secondary antibody provided by Dako, Carpinteria, CA, USA. Immunodetection was achieved by using a diaminobenzidine substrate kit (Vector Laboratories Inc., Burlingame, CA, USA). Hematoxylin counterstaining was carried out, then dehydration as well as xylene clearing. After that, tissue portions were covered to be examined under a microscope; According to method adopted from (Ibrahim et al. 2023) and (Mohamed et al. 2023). From each group's processed DRG samples, six non-overlapping areas were selected at random and scanned. For the histological analysis, the examiner histologist used the Leica Application module connected to the full HD microscopic imaging system “Leica Microsystems GmbH, Germany” to determine the mean positive reactive macrophage count of CD163- and CD86- in immunohistochemically stained sections.

### ELISA Assay for Biochemical Parameters

By the use of ELISA kits designed specifically for rats provided by Mybiosource (CA, USA), the spinal cord DRG components of the following markers: cAMP (Cat# MBS2700004), interleukin-4 (IL-4) (Cat# MBS355442), inducible nitric oxide synthase (i-NOS) (Cat# MBS263618), and arginase-1 (Cat# MBS917512) were established by following the guidelines provided by the relevant kit. Additionally, tumor necrosis factor-α (TNF- α) was quantified by Cusabio, (TX, USA) ELISA kit (Cat# CSB- E11987r). Tissue homogenates' protein content was assessed using the technique of (Bradford 1976).

### Western Blot Analysis

Using radioimmunoprecipitation assay buffer, DRG-isolated tissues were homogenized and lysed. A Bradford Protein Assay Kit (Bio BASIC INC., Ontario, Canada, Cat# SK3041) was then used to fully extract the proteins. After that, samples were loaded with Laemmli Buffer and then boiled for 5 min at 95 °C to make sure of protein denaturation (Hirano 2012). According to their molecular weight, the sample proteins were separated using SDS-PAGE and blotted on a polyvinylidene difluoride membrane. Tris-buffered saline with Tween 20 (TBST) buffer and 3% bovine serum albumin were used to block the polyvinylidene difluoride membrane for 1 h at room temperature to stop the primary and/or secondary antibodies from nonspecifically attaching to the membrane. TBST was used to dilute the primary antibodies, and they were then incubated at 4 °C for the entire night. The following used antibodies provided by Thermo Fisher Scientific, MA, USA were anti-β-actin (Cat# MA5-15739), anti-PKA (Cat# PA5-17626), and anti-p-CREB (Ser133) (Cat# PA1-4619). After incubating, the blots were rinsed 3 times using TBST for 5 min, incubated for one hour at room temperature with the matching secondary antibody solution conjugated with horseradish peroxidase, and then rinsed with TBST once more. To evaluate the band intensity of proteins, the ChemiDoc MP Imaging System “Bio-Rad Laboratories, CA, USA” was used. Normalization against the relevant values of β-actin was then carried out.

### Statistical Analysis

Statistical analysis was made using a one-way ANOVA followed by the Tukey’s multiple comparisons test. The data were shown as mean ± SD. However, for assessment of immunohistochemical expression of CD86 and CD163, Kruskal-Walli’s test followed by Dunn’s test were used and the results were displayed as median and range. The behavioral tests were performed using two-way ANOVA followed by the Tukey–Kramer multiple comparisons test. The statistical analysis was done using GraphPad Prism software (version 9), with a significance level of *P < *0.05.

## Results

### Semaglutide Mitigated Reserpine-induced Hyperalgesia in Rats

Three days of reserpine injection (day 0) induced a state of hyperalgesia that lasted throughout the investigational period. This was evidenced by demonstrating profound depression in paw withdrawal threshold (50.35%) and tail withdrawal latency (42.46%) in Randall-Selitto and tail immersion tests, respectively, as compared to the control group. Also, the hot plate test displayed a marked decline in thermal pain behavior latency to 29.74%, as compared to the control group.

Following the three days of reserpine injection, treatment with semaglutide at three different doses LD, ID, and HD for 14 days succeeded in ameliorating the hyperalgesia triggered by reserpine administration. This was indicated by restoring the values of hot plate and tail immersion tests to the control ranges. In Randall-Selitto test, Res-injected rats that were treated with semaglutide (LD, ID, and HD) for 14 days displayed marked rise in paw withdrawal threshold to 2.19-, 2.56-, and 3.33- folds, respectively, where only the HD semaglutide reinstated normal values.

**Fig. 1:**
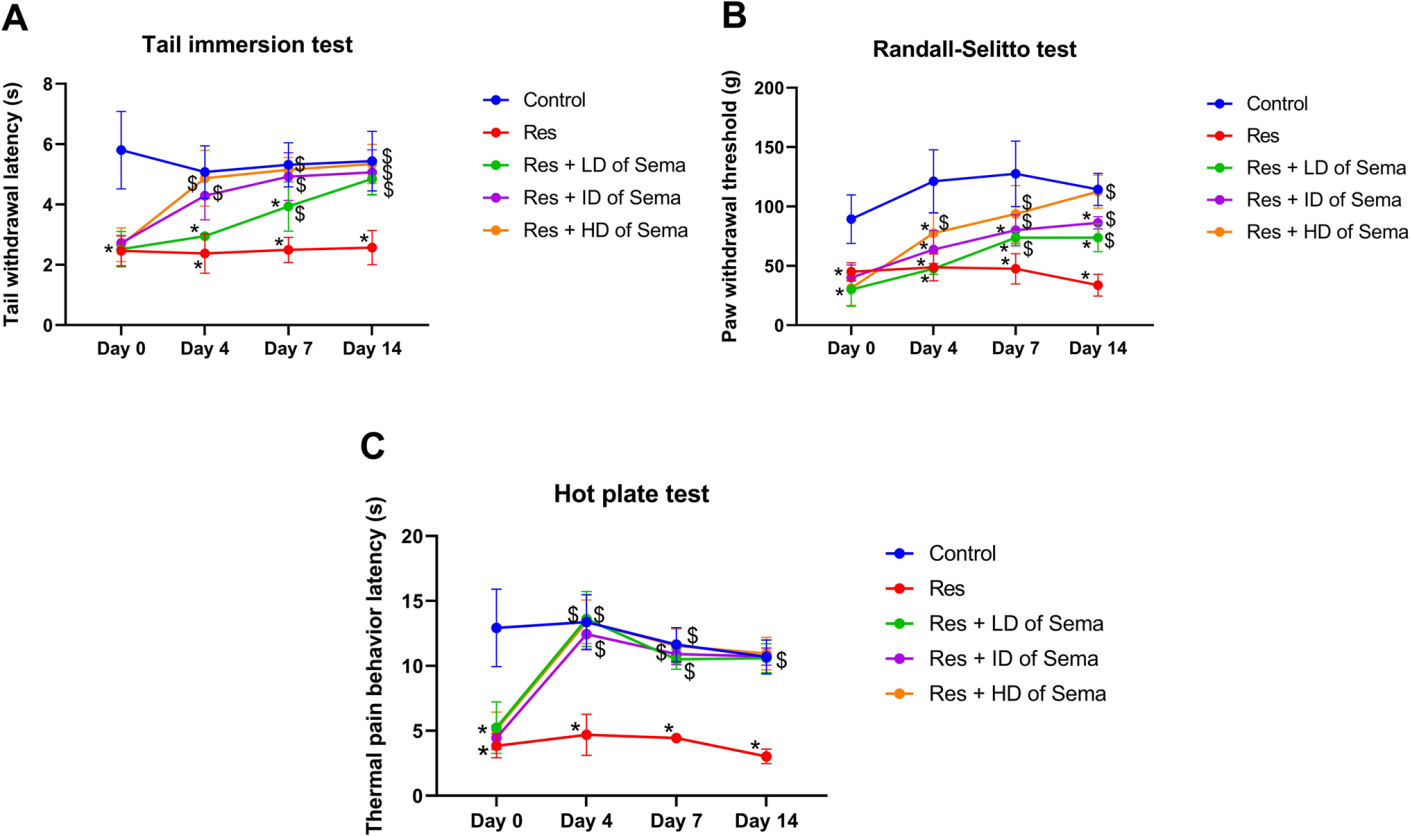
Effect of semaglutide on behavioral and hyperalgesia in reserpine rats. Panels represent withdrawal latency time (**A**) in tail immersion test, paw withdraw threshold (**B**) in Randall-Selitto test, and latency time (**C**) in hot plate test. Res reserpine, Sema semaglutide, LD low dose, ID intermediate dose, HD high dose. Each bar with vertical line represents mean ± S.D. (*n = *8) using two-way ANOVA followed by Tukey–Kramer multiple comparison post-test; *p < *0.05, ^*^ *vs* control group and ^$^ *vs* Res group

Noteworthy, the inhibitory effect of low, intermediate, and high doses of semaglutide on reserpine-induced hyperalgesia was evident on day 4 and 7 of its administration in hot plat test returning the thermal pain latency values to the range of their control counterparts. However, in tail immersion test, only ID and HD semaglutide counteracted reserpine-induced hyperalgesia after 4 days of their administration producing values insignificantly different from the control results. In Randall-Selitto test, only HD semaglutide was effective to reduce reserpine-induced hyperalgesia on day 4 of its administration significantly increasing the paw withdrawal threshold to 1.6- folds, reinstating the normal values on day 7 of its administration (Fig. 1).

### Semaglutide Improved Reserpine-induced Depressive Behavior and Motor-incoordination in Rats

Reserpine administration for three days (day 0) remarkably precipitated depressive-like symptoms and produced negative effect on motor functions as witnessed in forced swimming and rotarod tests during the tested four-points interval. Res-injected rats demonstrated a significant increase in immobility time contrary to profound depression in rotarod fall off latency to 3.24- folds and 23.63%, respectively, as compared with the control group.

Three days following reserpine injection, treatment with semaglutide at three different doses (LD, ID, and HD) for 14 days could amend the depressive state and ameliorate the motor dysfunction prompted by reserpine. This was shown by returning the values of immobility time and fall off latency to the control ranges.

**Fig. 2:**
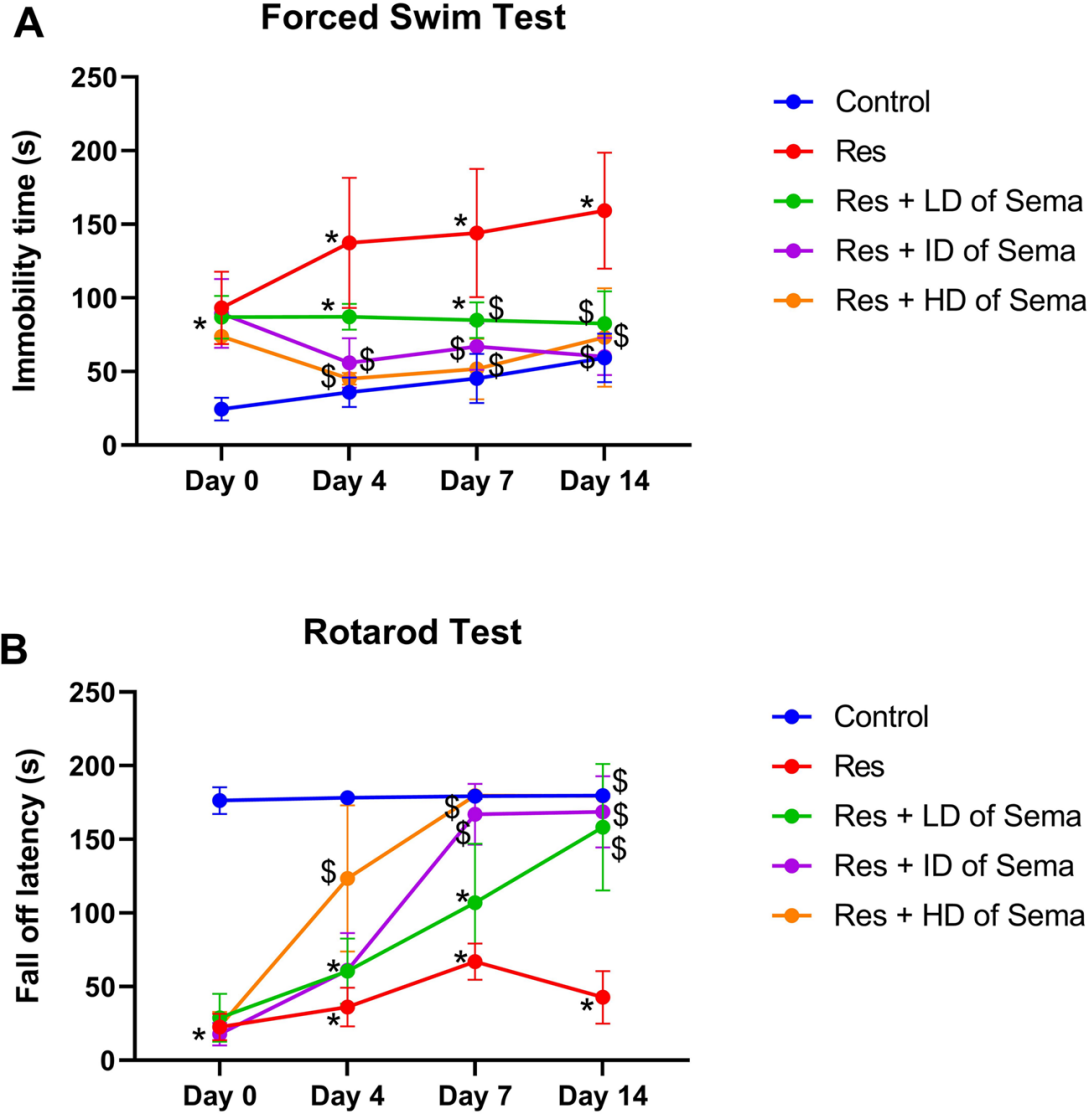
Effect of semaglutide on depressive behavior and motor alteration in reserpine rats. Immobility time (**A**) in forced swim test and fall off latency time (**B**) in rotarod test. Res reserpine, Sema semaglutide, LD low dose, ID intermediate dose, HD high dose. Each bar with vertical line represents mean ± S.D. (*n = *8) using two-way ANOVA followed by Tukey–Kramer multiple comparison post-test; *p < *0.05, ^*^ *vs* control group and ^$^ *vs* Res group

Interestingly, in the forced swim test, Res-injected rats that were treated with ID and HD of semaglutide only counteracted Res-induced depression after 4 days of their administration producing comparable values with the control results. In rotarod test, only HD of semaglutide was effective to alleviate Res-induced motor incoordination on day 4 of its administration, reinstating normal values. While ID semaglutide started to exert a significant enhancing effect on motor function on day 7 of its administration producing analogues results with those of the control group (Fig. 2).

### Semaglutide Attenuated the Changes in PKA/CREB Signaling Pathway Caused by Reserpine

**Fig. 3:**
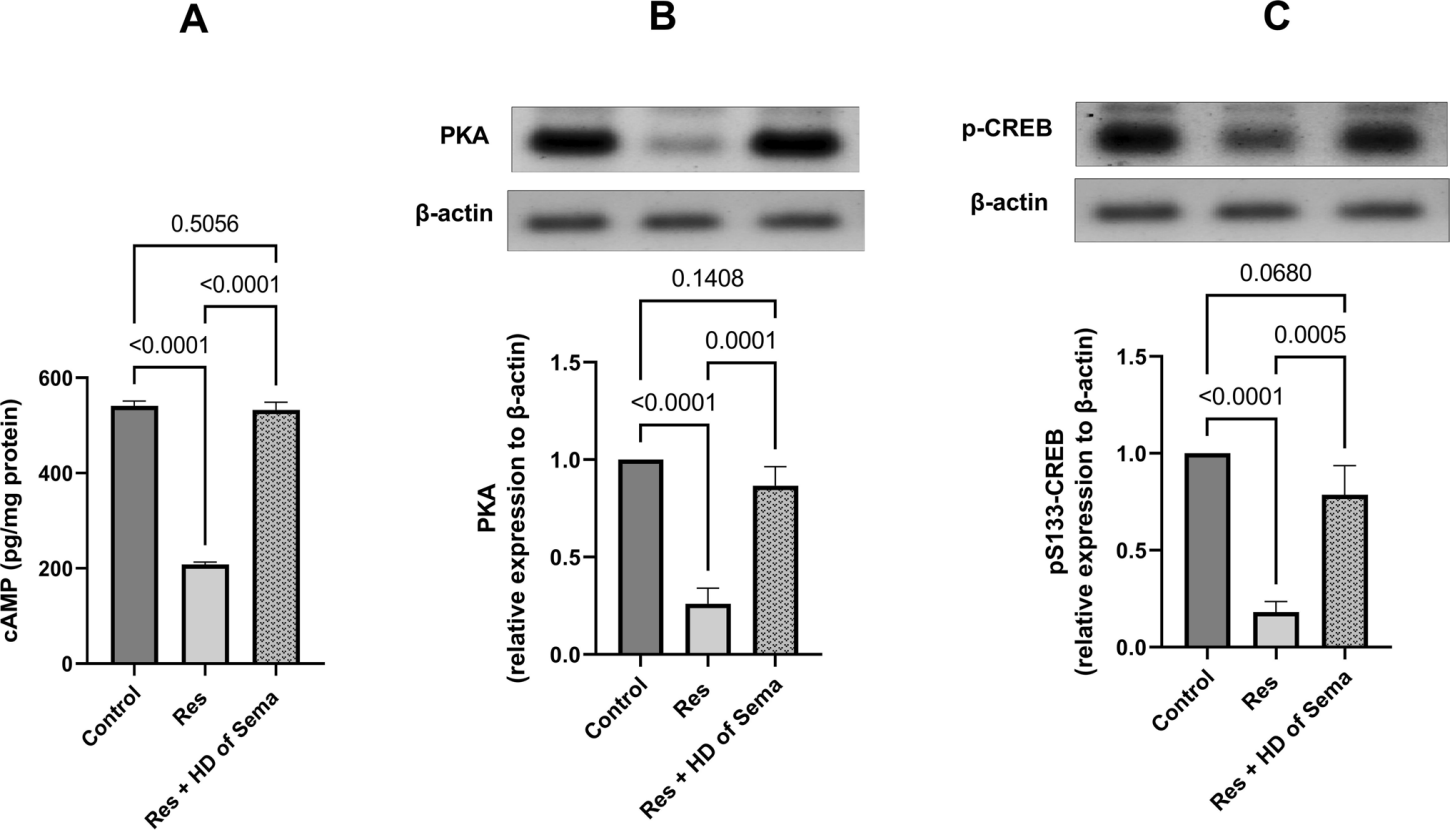
Effect of semaglutide on reserpine-induced depletion in spinal cord contents of cAMP (**A**), PKA (**B**), and P-CREB (**C**). Res reserpine, Sema semaglutide, HD high dose, PKA protein kinase A, p-CREB phosphorylated cAMP response element (CRE)-binding protein. Each bar with vertical line represents mean ± S.D. (*n = *5 for cAMP, while *n = *3 for PKA and p-CREB) using one-way ANOVA followed by Tukey–Kramer multiple comparison post-test; *p < *0.05

Reserpine-injected rats demonstrated a marked drop in amounts of PKA (26%), p-CREB (18%), and cAMP (38.48%) in the DRG of spinal cord as compared to the control group. Treatment of reserpine-injected rats with high dose of semaglutide raised the levels of PKA (3.33- folds), p-CREB (4.37- folds) and cAMP (2.56- folds), in comparison with Res group (Fig. 3).

### Semaglutide Mitigated the Dysregulation of M1/M2 Macrophage Polarization Induced by Reserpine

**Fig. 4:**
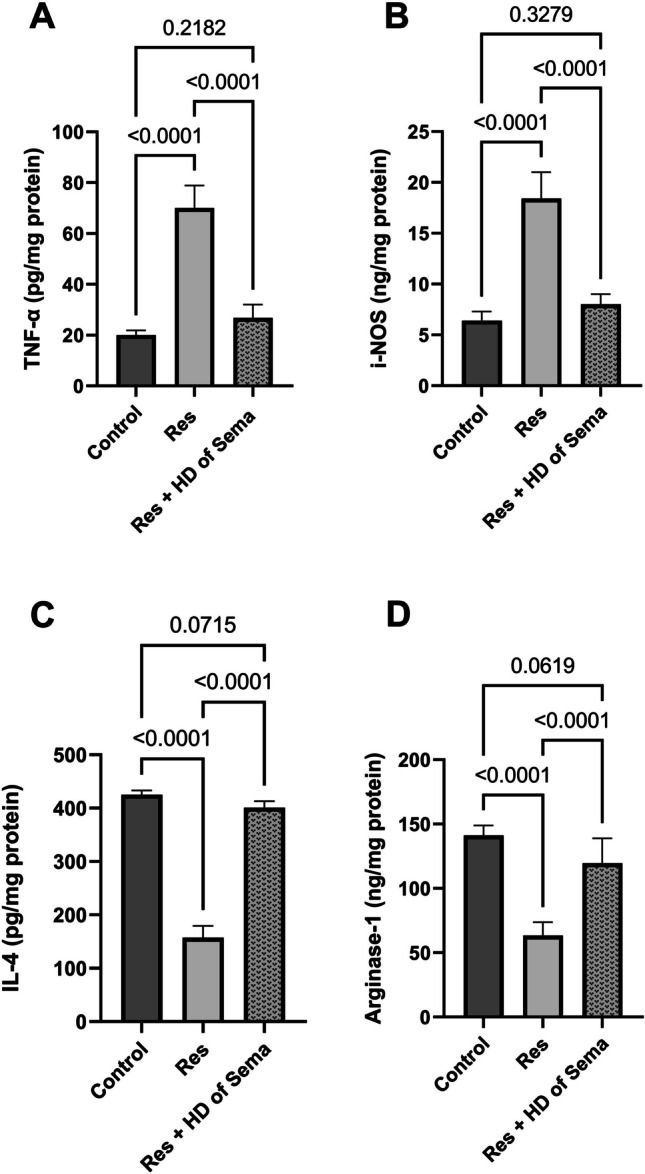
Effect of semaglutide on reserpine-induced depletion in spinal cord contents of TNF-α (**A**), i-NOS (**B**), IL-4 (**C**), and Arginase-1 (**D**). Res reserpine, Sema semaglutide, HD high dose, TNF-α tumor necrosis factor α, i-NOS inducible nitric oxide synthase, Il-4 interleukine-4. Each bar with vertical line represents mean ± S.D. (*n = *5) using one-way ANOVA followed by Tukey–Kramer multiple comparison post-test; *p < *0.05

The apparent inflammatory response to reserpine injection was attributed to a shift in M1/M2 microglia polarization towards M1. As compared to the normal group, the DRG concentrations of TNF-α and i-NOS were markedly elevated in Res group rats to 3.5- folds and 2.88- folds, respectively. Otherwise, the levels of arginase-1 and IL-4 were diminished in DRG to 44.9 and 36.99%, respectively, as compared to the normal rats. Significant anti-inflammatory effects were shown by a high dose of semaglutide, which caused the microglia polarization shifting to M2 as shown by lessening the concentrations of TNF-α and i-NOS to 38.29 and 43.48%, respectively, in contrast to elevating arginase-1 and IL-4 to 1.89- and 2.55- folds, respectively, in comparison with reserpine group (Fig. 4).

**Fig. 5:**
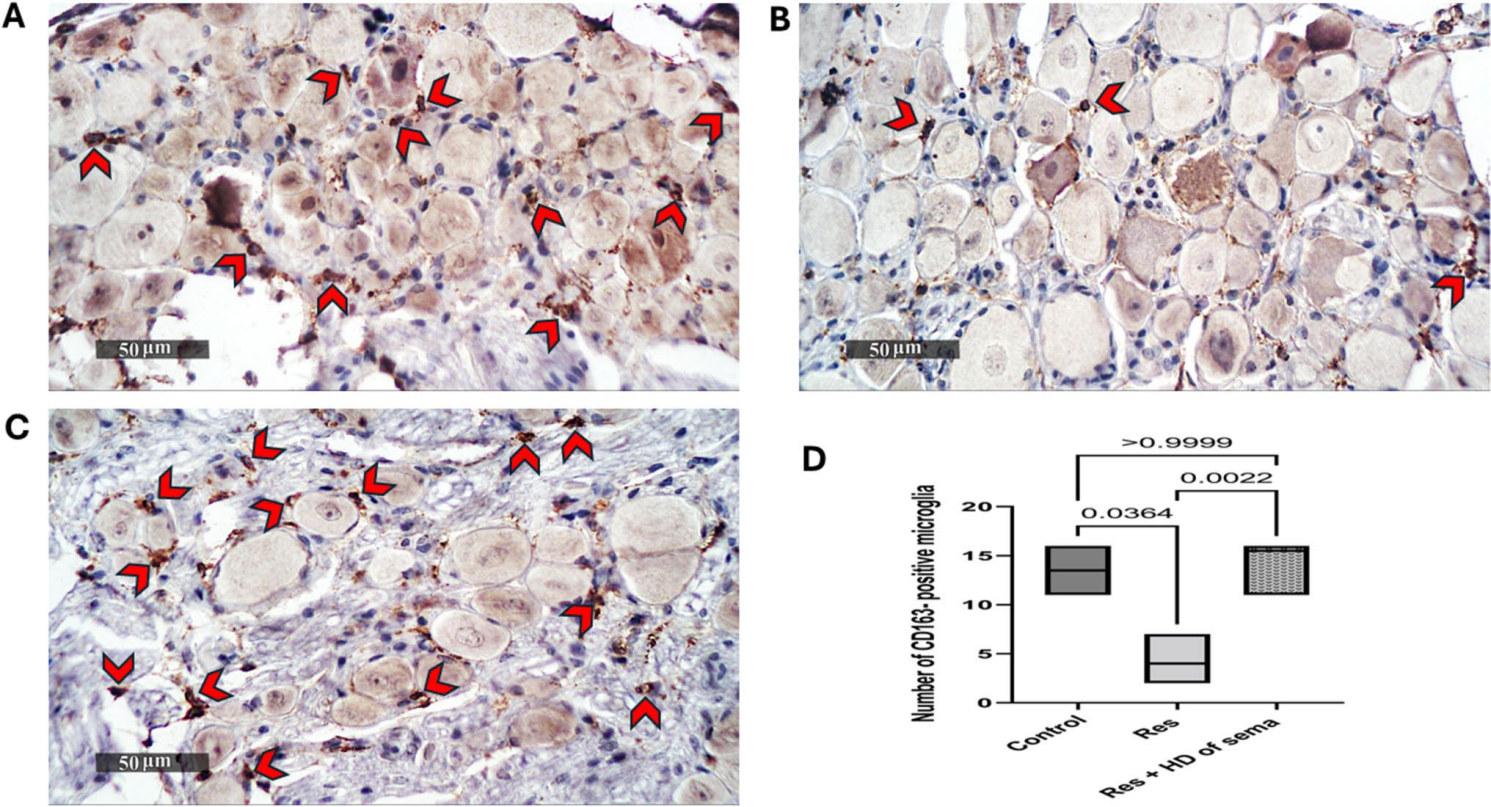
Effect of semaglutide on reserpine-induced alteration in spinal cord immuno-histochemical expression of CD163. Control group (**A**), Res group (**B**), and Res + HD of Sema group (**C**). Magnification: 50 μm. *red arrow heads* point for CD163-positive microglia. **D** Bar chart represents the mean count of CD163-positive microglia where each bar with vertical line represents median and range (*n = *3) using Kruskal-Walli’s test followed by Dunn’s test; *p < *0.05. Res reserpine, Sema semaglutide, HD high dose, CD163 cluster of differentiation 163

**Fig. 6:**
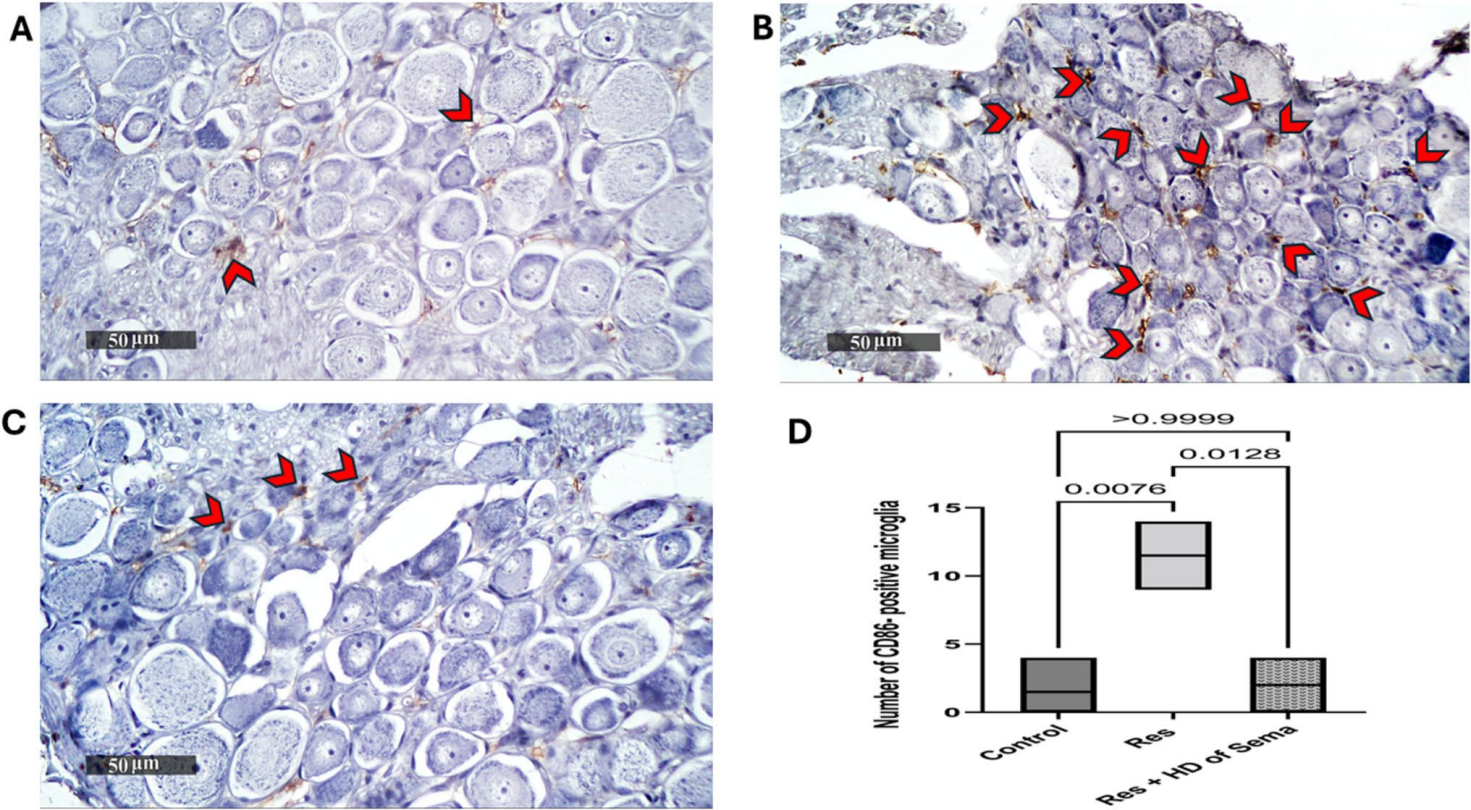
Effect of semaglutide on reserpine-induced alteration in spinal cord immuno-histochemical expression of CD86. Control group (**A**), Res group (**B**), and Res + HD of Sema group (**C**). Magnification: 50 μm. *red arrow heads* point for CD86-positive microglia. **D** Bar chart represents the mean count of CD86-positive microglia where each bar with vertical line represents median and range (*n = *3) using Kruskal-Walli’s test followed by Dunn’s test; *p < *0.05. Res reserpine, Sema semaglutide, HD high dose, CD86 cluster of differentiation 86

CD86 is a costimulatory ligand expressed mainly by monocytes/macrophages. However, CD163 macrophages play an anti-inflammatory role. In the present data, reserpine-injected rats demonstrated extensive upregulation of DRG immunohistochemical expression of CD86 in comparison with the normal group. Meanwhile, the immunoreactivity of CD 163 was remarkably downregulated in DRG of reserpine group in comparison with the normal group. Interestingly, high dose of semaglutide displayed a remarkable rise in immunohistochemical expression of CD163 contrary to a significant diminution in CD86 level as compared with reserpine group (Figs. 5 and 6).

### Semaglutide Reduced the Histopathological Alterations in the Spinal Cord Caused by Reserpine

**Fig. 7:**
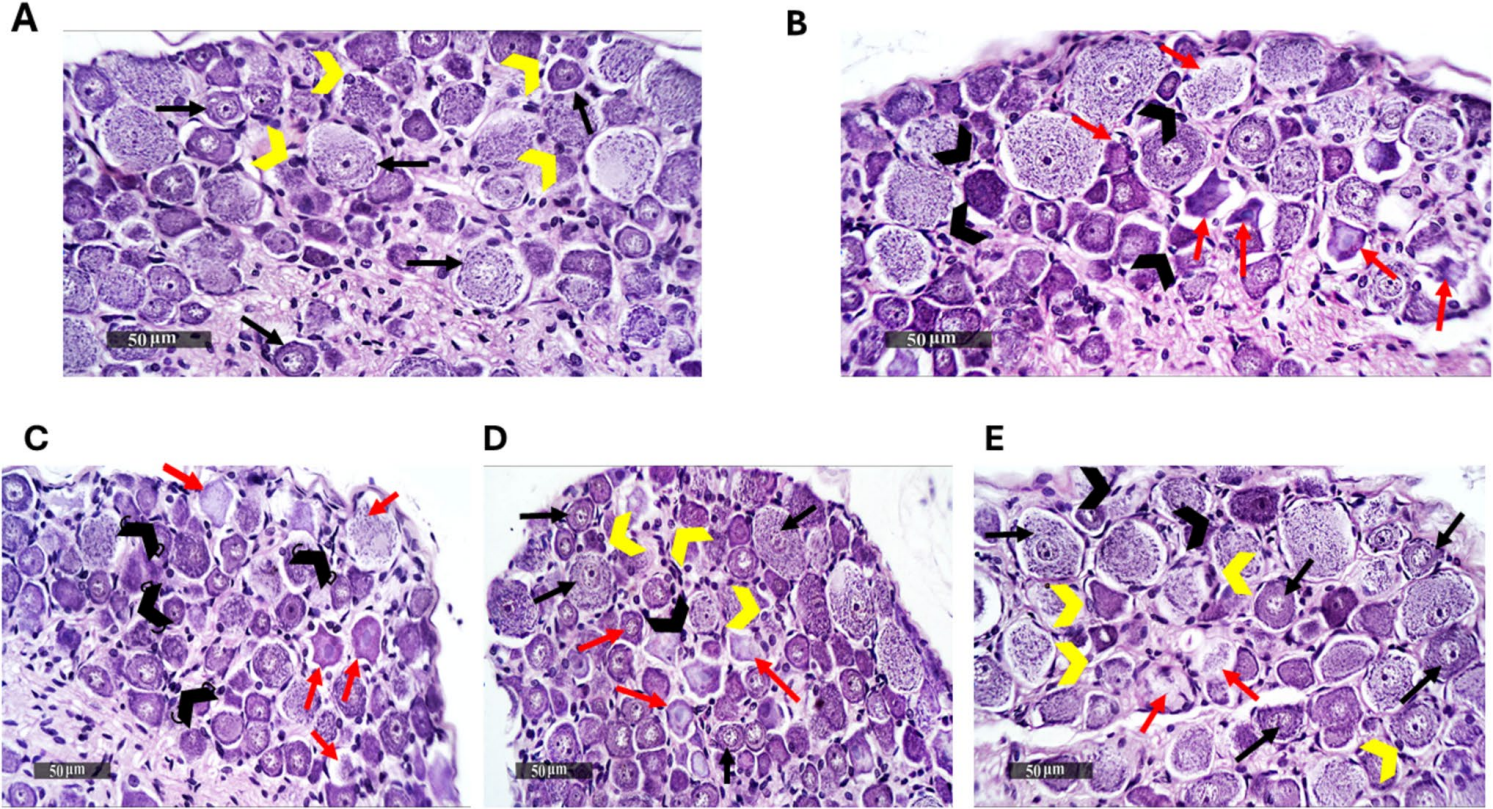
Effect of semaglutide on reserpine-induced histopathological alterations in rats’ spinal cord.Control group (**A**), Res group (**B**), Res + LD of sema group (**C**), Res + ID of Sema group (**D**), Res + HD of Sema group (**E**). Representative H&E photomicrographs of all experimental groups (*n = *3). Magnification: 1 mm and 50 μm. *Black arrows* indicate intact small and large neuronal types with intact subcellular details, *yellow arrowheads* indicate normal organized satellite ganglionic cells, *red arrows* indicate focal dispersed records of chromatolysis as well as shrunken angular neurons with nuclear pyknosis, *black arrowheads* indicate figures of mononuclear cells infiltrates – mainly macrophages with a marked perineural ganglionic satellite cells loss

Numerous figures of seemingly intact small and big neuronal types with intact subcellular details (black arrow) were displayed by the control group, which displayed normal ordered histological features of DRG. In addition, it illustrated minimal sporadic degenerated neurons, and normal organized satellite ganglionic cells (yellow arrowhead) as well as Schwann cells with intact myelinated nerve fibers. On the other hand, reserpine group samples exhibited focal dispersed records of chromatolysis as well as shrunken angular neurons with nuclear pyknosis (red arrow). They also presented moderately higher figures of mononuclear cells infiltrates – mainly macrophages (black arrowhead) with a marked perineural ganglionic satellite cells loss. Samples of Res + LD of Sema group revealed similar records as reserpine group samples. However, Res + ID of Sema group samples showed a higher widespread figure of apparent intact neurons (black arrow) with persistent limited occasional figures of neuronal damage and loss (red arrow). Moreover, higher densities of more organized satellite ganglionic cells (yellow arrowhead) with minimal records of abnormal mononuclear cells infiltrates (black arrowhead) were observed. Similarly, Res + HD of Sema group samples demonstrated nearly the same records as those presented by the intermediate dose of semaglutide samples (Fig. 7).

## Discussion

This research provided the first evidence of the therapeutic benefits of semaglutide in a reserpine rat model of FM-like symptoms. This concept is reinforced by (1) improvement of sensory and motor behavioral deficits, (2) diminution in histopathological changes in the spinal cord, (3) alleviation in hyperalgesia, (4) reduction of inflammation through inhibition of i-NOS, (5) activation of the PKA/CREB pathway to improve neuroprotection and cell survival, (6) amelioration of inflammation in neurons via increasing the anti-inflammatory markers of M2 microglia phenotype.

Here, the nociception anomalies caused by reserpine injection were demonstrated using Randall-Selitto, tail immersion, and hot plate tests. Nonetheless, semaglutide treatment drastically alleviated the effects of reserpine-induced hyperalgesia, exhibiting an impressive rise in thermal pain behavior latency, tail withdrawal latency, and limb withdrawal threshold. It was demonstrated that exenatide, a GLP-1R agonist, via activating the spinal dorsal horn GLP-1Rs, effectively blocked pain hypersensitivity and induced beta-endorphin release with subsequent activation of opioid receptors producing antinociception in pain hypersensitivity states (Gong et al. 2014).

The main reason of the motor impairment and incoordination detected in rats exposed to reserpine was the depletion of central and peripheral monoamines, such as serotonin, dopamine, and norepinephrine (Yao et al. 2019). Here, semaglutide enhanced reserpine-injected rats' motor coordination, as seen by the longer time the rats spent gripping the revolving rod owing to increasing the serotonin level in CNS (de Paiva et al. 2023).

Reserpine administered rats in the current study showed signs of depression, including an obvious rise in time remaining immobile during a forced swimming test, in harmony with prior studies (Brum et al. 2020; Atta et al. 2023). Additionally, they demonstrated a significant drop in PKA, p-CREB, and cAMP concentrations in comparison with the normal group, similar to previous researches (Atta et al. 2023; Shafiek et al. 2024). Oxidative stress along with inflammation caused CREB expression to be downregulated, which impaired neurogenesis and resulted in the development of depressed behaviors (Ge et al. 2015). Furthermore, in the rodents' chronic restraint stress FM model, depression and hyperalgesia have been related to low levels of p-CREB (Lee et al. 2017). Herein, semaglutide ameliorated depressive-like behavior provoked by reserpine producing a significant reduction in immobility time recorded in forced swim test. This potential antidepressant effect of semaglutide is attributed to being a GLP-1R agonist causing stimulation of adenylyl cyclase and consequently cAMP. The latter causes exchange protein activated by cAMP, Epac, to become active, which has subsequent antiapoptotic and anti-inflammatory impacts (Tamayo-Trujillo et al. 2024). cAMP activates PKA, which in turn activates MAPK/ERK anti-inflammatory signaling and also activates and phosphorylates the transcription factor CREB (Jolivalt et al. 2011). The latter is a crucial marker for neurogenesis and neuronal survival, and several neuro-pathophysiological effectors are responsive to its neuroprotective, anti-apoptotic, and antioxidant properties (Sakamoto et al. 2011).

Neuroinflammation is a widespread characteristic of many neurological illnesses and chronic pain conditions (Colloca et al. 2017; Uniyal et al. 2022; Gadepalli et al. 2024; Singh et al. 2024). It is a significant pathogenic element in FM, particularly when it is mediated by microglia (Song and Suk 2017; Seo et al. 2021). The development of neuroinflammatory diseases is significantly influenced by macrophages and microglia, which modify neuroinflammatory reactions in both positive and negative manners. There are two distinct macrophage functionally active phenotypes: classically activated macrophages “M1, pro-inflammatory, neurotoxic” and alternatively-activated macrophages “M2, anti-inflammatory, neuroprotective)” (Kettenmann et al. 2013; An et al. 2021). M1 microglia release inflammatory molecules, reactive oxygen species (ROS), chemokines, prostaglandin E2, and cytokines, including interleukin-1β (IL-1β) and TNF-α; which can harm neurons when they are present in excess by triggering intracellular inflammatory pathways that include cyclooxygenases 1 and 2, ROS, and nuclear factor kappa-B among other signaling molecules (Shabab and Zorofchian 2016). There are extra ramifications of neuroinflammation that involve more oxidative stress, DNA damage, and rises in ROS. Other cytokines have anti-inflammatory properties and work against these inflammatory reactions to maintain homeostatic regulatory control. There is an issue with a significant imbalance between cytokines that promote and inhibit inflammation. Inflammatory-promoting cytokine TNF-α triggers apoptosis by activating several receptors, such as TNF receptors and CD 95. On the other hand, IL-1β triggers the IL-1 receptor, which triggers signaling cascades that result in inflammation and excitotoxicity as well as neurodegeneration (Shabab and Zorofchian 2016). Numerous TNF-α inhibitors have been investigated in rat models of central nervous system disorders and have been shown to boost cognitive performance, preserve synapses, reduce apoptosis, promote neuronal survival, and minimize neuroinflammation (Chen et al. 2016; Lin et al. 2020), suggesting that high TNF-α activation has a negative impact on neuronal survival and CNS health. Injecting IL-1β into the rat striatum elevated the expression of adhesion molecules, disrupted the BBB, and enhanced i-NOS, which in turn facilitated the diffusion of damaging nitric oxide (NO) (Blamire et al. 2000). Furthermore, pro-inflammatory cytokines raise the synthesis of i-NOS, which in turn elevates NO levels that has several neurotoxic pathways, such as peroxynitrite generation, DNA damage, glutamate excitotoxicity, and apoptosis signaling cascade activation (Dawson and Dawson 2018). Oxidative stress and ROS co-occur with neuroinflammation and T2DM (Hauck et al. 2019). It has been discovered that exenatide treatment reduced NO, a sign of inflammatory marker, in atherosclerosis by inhibiting i-NOS (Bułdak et al. 2015).

It is believed that M1 modulation activates superficial layers of the motor cortex, which sets in motion a sequence of synaptic events that modulate activity throughout a vast neuronal network, including the brainstem nuclei, spinal cord, limbic system, and thalamic nuclei. It is believed that these locations are crucial to the pain matrix (Khedr et al. 2017). Elevated expression of M1 macrophage-specific molecules and reduced expression of M2 macrophage-specific molecules support the shifting of M1/M2 macrophage polarization in the direction of the inflammatory M1 phenotype in a variety of neuropathic pain types (Tripathi et al. 2021). The precipitation of nociplastic pain in FM patients is caused by M1 macrophage-dependent neuroinflammation (Atta et al. 2023; Tripathi et al. 2021).

In the current study, M1 reactions were tracked by measuring the spinal cord concentrations of CD86 and i-NOS, whereas M2 responses were tracked by measuring the spinal levels of CD163 and arginase-1 (Moehle and West 2015; An et al. 2021). The present findings demonstrated that the reserpine group had inflammatory responses as evidenced by elevated levels of CD86 and i-NOS. However, arginase-1 levels were low and CD163 immunoreactivity was reduced. On the other hand, semaglutide treatment exerted anti-inflammatory outcomes showed by reduced i-NOS and CD86 as well as markedly elevated arginase-1 and CD163. Additionally, semaglutide demonstrated neuroprotective benefits to the CNS leading to enhancement to cell survival, decreased M1-like microglia numbers, and reduced interleukin-6 and TNF-α levels in different brain areas in obesity (Tamayo-Trujillo et al. 2024). Further, semaglutide stimulated the anti-inflammatory markers of M2 microglia subset and the cAMP/PKA/p38β pathway, which in turn stimulated the expression of cell-specific anti-inflammatory factors, interleukin-10 and IL-4 (Tamayo-Trujillo et al. 2024).

## Conclusion

The current study sheds light on semaglutide's FM-ameliorating effects. It corrected the neuroinflammation, nociplastic pain, and spinal cord histological changes caused by reserpine. It stimulates cAMP/PKA pathway that causes an increase in M2 microglia polarization markers. Therefore, the present study offers a novel perspective on the therapeutic impact of semaglutide in FM. However, further studies are recommended to compare semaglutide’s effects in FM with those afforded by the officially approved drugs for FM which are amitriptyline, pregabalin and duloxetine (Kia and Choy 2017). Further, it is encouraged to investigate the influence of semaglutide on other markers of M1/M2 macrophage polarization.

## Supplementary Information

Below is the link to the electronic supplementary material.

Supplementary file1 (PDF 68 KB)

## Data Availability

No datasets were generated or analysed during the current study.

## Abbreviations

BBB: Blood brain barrier
cAMP: Cyclic adenosine monophosphate
CD163: Cluster of differentiation 163
CD86: Cluster of differentiation 86
CREB: CAMP response element (CRE)-binding protein
DM: Diabetes mellitus
DRG: Dorsal root ganglia
ERK: Extracellular signal-regulated kinase
FM: Fibromyalgia
GLP-1: Glucagon-like peptide-1
GLP-1R: Glucagon-like peptide-1 receptor
HD of Sema: High dose of semaglutide
IR: Insulin resistance
i-NOS: Inducible nitric oxide synthase
ID of Sema: Intermediate dose of semaglutide
IL-4: Interleukin-4
IL-1β: Interleukin-1β
IL-1R: Interleukin -1 receptor
LD of Sema: Low dose of semaglutide
M1: Macrophage 1
M2: Macrophage 2
MAPK: Mitogen activated protein kinase
NO: Nitric oxide
PKA: Protein kinase A
Res: Reserpine
ROS: Reactive oxygen species
Sema: Semaglutide
TBST: Tris-buffered saline with Tween 20
TNF- α: Tumor necrosis factor-α
TNFR: Tumor necrosis factor receptor
