# Supplementary material for: Novel Trajectories Towards Possible Effects of Semaglutide for Amelioration of Reserpine-induced Fibromyalgia in Rats: Contribution of cAMP/PKA/p-CREB and M1/M2 Microglia Polarization
Source: J Neuroimmune Pharmacol. 2025 Apr 17;20(1):43. doi: 10.1007/s11481-025-10196-4 (PMC12003577; doi:10.1007/s11481-025-10196-4)
Supplement: Supplementary file 1 — Supplementary file1 (PDF 68 KB) [file 11481_2025_10196_MOESM1_ESM.pdf]

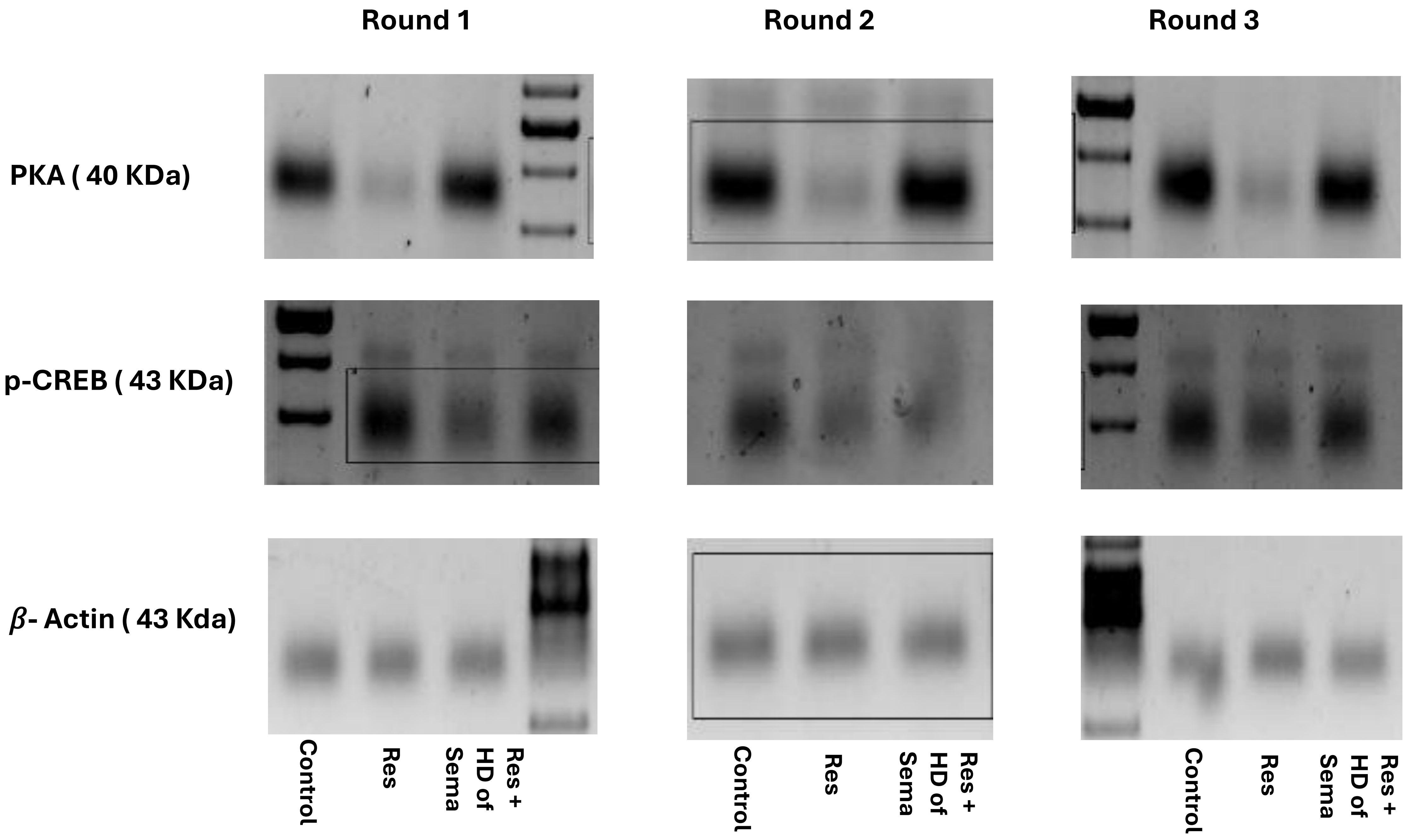
Round 1
Round 2
Round 3
PKA (40 KDa)
p-CREB (43 KDa)
β- Actin (43 Kda)
Control
Res
Sema
HD of
Res +
